# The Effect of Preoperative Hematocrit Level on Early Outcomes After Coronary Artery Bypass Surgery

**DOI:** 10.7759/cureus.7811

**Published:** 2020-04-24

**Authors:** Arda Aybars Pala, Temmuz Taner, Ahmet Burak Tatli, Kadir Kaan Ozsin, Senol Yavuz

**Affiliations:** 1 Cardiovascular Surgery, Adıyaman Training and Research Hospital, Adıyaman, TUR; 2 Cardiovascular Surgery, University of Health Sciences, Bursa Yüksek İhtisas Training and Research Hospital, Bursa, TUR

**Keywords:** coronary artery bypass grafting(cabg), hematocrit, mortality

## Abstract

Introduction: Low hematocrit level is a hematological problem that is frequently encountered in the preoperative evaluation of patients undergoing coronary artery bypass grafting (CABG) surgery. The aim of this study was to investigate the effect of preoperative hematocrit level on the first 30-day outcomes in patients undergoing CABG surgery.

Methods: Ninety-four patients undergoing isolated CABG were included in the study. The patients were divided into two groups as patients with preoperative low hematocrit levels (<36%) in Group 1 and patients with preoperative normal hematocrit levels (≥36%) in Group 2.

Results: Forty-six patients in Group 1 (mean age: 63.6 ± 7.9 years) and 48 patients in Group 2 (mean age: 56.5 ± 8.8 years) were enrolled. European System for Cardiac Operative Risk Evaluation (EuroSCORE) scoring was statistically significantly higher in Group 1 (p = 0.011). In the postoperative period, the amount of drainage, transfusion of blood, and blood products were significantly higher in Group 1 (p < 0.001). The mortality rate of Group 1 was statistically higher in the first 30 days postoperatively (p = 0.020).

Conclusion: Low preoperative hematocrit levels are associated with increased mortality after CABG surgery. We suggest that patients’ preoperative hematocrit levels must be added to the risk scoring systems as an assessment parameter.

## Introduction

Coronary artery disease (CAD) is the most important part of cardiovascular diseases [1]. CAD is spreading all over the world and reaching life-threatening dimensions. Coronary artery bypass grafting (CABG) is the most effective method for the treatment of ischemic heart disease. In some studies, it has been reported that patients with high-risk scores have better benefits from CABG surgery [2]. For the last 10 years, many studies have been done to determine the surgical risk factors of CAD and preoperative risk scoring has become extremely important in patients undergoing CABG surgery. The success of surgery depends to some extent on the elimination or improvement of these factors, or on taking measures against it [3,4]. Some of these factors include advanced age, poor ventricular function, presence of diffuse coronary lesions, presence of poor respiratory or renal function, previous cardiac surgery, complicated surgery, and emergent surgical intervention. The aim of this study was to investigate whether the hematocrit level, which is routinely assessed in the preoperative period, is a parameter affecting clinical outcomes in the early postoperative period (first 30 days).

## Materials and methods

Ninety-four patients who underwent CABG operation between January 2011 and January 2015 were included in the study. Patient data were obtained retrospectively from the hospital registry system. Local ethics committee approval was obtained.

In our study, patients who underwent isolated CABG surgery in our hospital were screened according to hematocrit levels and those with low preoperative hematocrit levels were examined. The control group consisted of another group of patients who were randomly selected from patients who had normal preoperative hematocrit levels. The factors affecting the morbidity and mortality in the postoperative 30-day period were investigated by using the patient files.

The study included 46 patients undergoing CABG surgery with hematocrit levels lower than 36%, identified as Group 1, and 48 patients who underwent CABG surgery with normal preoperative hematocrit levels (36% and above), identified as Group 2. Demographic data, perioperative data, cardiac history, postoperative early morbidity and mortality results were evaluated. Ventricular functions were examined via transthoracic echocardiography on the day before surgery. The European System for Cardiac Operative Risk Evaluation (EuroSCORE) (logistic) scoring system was used for preoperative mortality risk assessment [5].

Patients with a previous history of CABG surgery, patients requiring additional surgical intervention other than CABG, patients who had to undergo emergency surgery due to hemodynamic instability, and patients with chronic kidney disease that can cause anemia and therefore increase the morbidity and mortality of CABG surgery were excluded from the study.

Surgical technique

Midazolam 0.003 mg/kg, fentanyl 5 pg/kg, and vecuronium bromide 0.1 mg/kg were used for induction of anesthesia. For maintenance of anesthesia, 5 pg/kg fentanyl and 0.5-1 mg/kg rocuronium or vecuronium bromide were used. All patients underwent a standard median sternotomy to reach the mediastinum. The left internal thoracic artery and saphenous vein grafts were prepared, considering the number of vessels to be operated and the grafts to be used. Cardiopulmonary bypass (CPB) was performed by using two-stage venous cannulation of the right atrial auricula, as a result of the planned procedure, and arterial cannulation through the ascending aorta. A cardioplegia cannula was placed in the aortic root and cold crystalloid cardioplegia was given to all patients initially. Myocardial protection with cold blood cardioplegia was performed. Warm blood cardioplegia was given for the purpose of preventing any reperfusion injury before the aortic cross-clamp was removed. Moderate hypothermia (30°C) was applied during the operation. A roller pump and membrane oxygenator were used in all cases. The pump flow was maintained between 2.2 and 2.4 L/min/m^2^ and was non-pulsatile to ensure the mean arterial pressure was maintained at 50-70 mmHg during the aortic cross-clamp interval. Hematocrit levels were kept between 20% and 25% during CPB. After removing the cross-clamp, heating was performed until the temperature of the bladder reached 36°C. After appropriate blood pressure and cardiovascular stability, the CPB was terminated. The patients were followed up in the intensive care unit in the postoperative period. Patients with a normal clinical course were transferred to a unit bed after their cannula was removed, which usually occurred on the second day postoperatively.

Statistical method

Statistical Package for the Social Sciences 16.0 (SPSS Inc., Chicago, IL) program was used to evaluate the data obtained from the study. Continuous variables (demographic, preoperative, perioperative, and postoperative data) were expressed as the mean ± standard deviation and categorical variables expressed as the percentage. Statistically normalized distribution was evaluated with Student's *t*-test and Mann-Whitney *U* test was used for non-normal distribution. Pearson's chi-squared test was used to evaluate categorical data. p values that were less than 0.05 were considered as statistically significant.

## Results

A total number of 46 patients were in Group 1 (73.9% male, mean age: 63.6 ± 7.9 years) and 48 patients were in Group 2 (93.8% male, mean age: 56.5 ± 8.8 years), which were recorded throughout the study.

The demographic and clinical properties of the subjects are summarized in Table 1. Both Group 1 and Group 2 were similar to each other in regard to demographic features. However, there were statistically significant differences between the two groups in terms of chronic obstructive pulmonary disease and EuroSCORE scores (p = 0.011).

**Table 1:** Comparison of demographic and preoperative data *t*: Student's *t*-test, m: Mann-Whitney *U* test, X²: chi-squared test (Fischer test) DM, diabetes mellitus; COPD, chronic obstructive pulmonary disease; HT, hypertension; CVE, cerebrovascular events; AF, atrial fibrillation; EF, ejection fraction; Hb: hemoglobin; Hct: hematocrit

	Group 1 (n = 46)	Group 2 (n = 48)	p
	Mean ± SD/n (%)	Mean ± SD/n (%)	
Age	63.6 ± 7.9	56.5 ± 8.8	0.489^t^
Sex (male, female)	34 (73.9%); 12 (26.1%)	45 (93.7%); 3 (6.3%)	0.148^X²^
DM	25 (54.3%)	20 (41.7%)	0.219^X²^
COPD	16 (34.8%)	6 (12.5%)	0.011^X²^
HT	37 (80.4%)	37 (77.1%)	0.691^X²^
CVE	1 (2.2%)	1 (2.1%)	0.976^X²^
Preoperative AF	2 (4.3%)	1 (2.1%)	0.532^X²^
EF	45.3 ± 10.7	44.4 ± 8.1	0.667^m^
EuroSCORE	3.7 ± 2	2.7 ± 1.7	0.011^m^
Preoperative Hb (g/dL)	10.1 ± 1	13.4 ± 1.5	0.039^m^
Preoperative Hct (%)	30.6 ± 2.3	39.4 ± 3	0.027^m^

Perioperative data included variables such as number of grafts placed during surgery, cross-clamp time, CPB time, intra-aortic balloon pump (IABP) support needs, and atrial fibrillation (AF) development between the groups. There were no statistically significant differences between groups in terms of number of grafts used (p = 0.843), cross-clamp time (p = 0.972), and CPB time (p = 0.597). The rate of AF development (p = 0.214) and need for IABP support (p = 0.175) during the perioperative and postoperative periods revealed no statistically significant difference between the groups (Table 2).

**Table 2:** Perioperative variables and distribution of data between groups m: Mann-Whitney *U* test, X²: chi-squared test (Fischer test) CPB, cardiopulmonary bypass; AF, atrial fibrillation; IABP, intra-aortic balloon pump

	Group 1 (n = 46)	Group 2 (n = 48)	p
	Mean ± SD/n (%)	Mean ± SD/n (%)	
Number of grafts used	3.13 ± 1	3.2 ± 0.9	0.843^m^
Cross-clamp time (minute)	66.1 ± 27	72.3 ± 24.5	0.972^m^
CPB time (minute)	90.1 ± 34.8	98.3 ± 32.5	0.597^m^
Perioperative and postoperative AF development	14 (30.4%)	12 (25%)	0.214^X²^
Perioperative and postoperative IABP support need	11 (23.9%)	9 (18.8%)	0.175^X²^

The comparison of postoperative parameters is shown in Table 3. In the postoperative period, signiﬁcant differences were observed between the two groups in terms of the amount of blood drainage during a 24-hour interval and blood and blood product transfusions (p < 0.001). Twenty-four hours of drainage amount was 680.43 ± 327.09 cc in Group 1 and 533.33 ± 390.35 cc in Group 2. In parallel to this, the amount of blood products (whole blood, erythrocyte suspension, and fresh frozen plasma) transfused in the 48-hour period was 3.3 ± 1.7 units in Group 1 and 1.9 ± 0.9 units in Group 2. When the groups were evaluated in terms of reoperation for bleeding, there was no statistically significant difference (p = 0.068). There was no significant difference between the two groups in terms of the duration of postoperative mechanical ventilation, length of stay in the intensive care unit, length of hospital stay, and infective complications.

**Table 3:** Comparison of postoperative data m: Mann-Whitney *U* test, X²: chi-squared test (Fischer test) ICU, intensive care unit

	Group 1 (n = 46)	Group 2 (n = 48)	p
	Mean ± SD/n (%)	Mean ± SD/n (%)	
Mechanical ventilation duration (hour)	13.5 ± 7	12.5 ± 4.7	0.398^m^
Drainage amount (cc)	680.4 ± 327	533.3 ± 390.3	<0.001^m^
Blood and blood product transfusion (unit)	3.3 ± 1.7	1.9 ± 0.9	<0.001^m^
Reoperation for bleeding	5 (10.8%)	4 (8.3%)	0.068^X²^
Length of stay in the ICU (day)	5 ± 3.5	3.3 ± 2.5	0.241^m^
Length of hospital stay (day)	18.8 ± 11.5	17.8 ± 5.7	0.580^m^
Infective complications	11 (23.9%)	6 (12.5%)	0.129^X²^
Mortality (in postoperative 30 days)	3 (6.5%)	1 (2.1%)	0.020^X²^

When the groups were evaluated in terms of mortality in the first 30 days postoperatively, mortality was observed in four patients. In Group 1, two patients died due to multiorgan dysfunction and one patient died due to low cardiac output. In Group 2, one patient died due to multiorgan dysfunction. The difference in in-hospital mortality rates in both groups was statistically significant (p = 0.020) (Table 3).

## Discussion

In our study, we assessed the effect of preoperative hematocrit levels on early outcomes after CABG surgery. We found that the mortality rate, postoperative drainage, and blood and blood product transfusion were statistically significantly higher in the group with low hematocrit levels.

Despite all the advances seen in heart surgery, morbidity and mortality seen after CABG surgery is still an important problem [6]. Due to the increased life expectancy, heart disease and associated mortality and morbidity rates also increase in older individuals. Considering this, the evaluation of this patient group is highly important to determine the preoperative risks. This fact increases the need for risk scoring systems. Because of the increase in the complication rate during and after surgery, there occurs an increase in cost and prolongs the length of hospital stay [7,8]. In the preoperative period, by using risk scoring systems, surgeon, anesthesiologist, intensive care unit team, the patient and the patient's relatives are more aware of what awaits them. In addition, risks may be reduced by applying intensive preoperative treatments to risk factors affecting morbidity and mortality [9].

In a previous study, Karabulut et al. evaluated 1,123 patients using the EuroSCORE scoring system [10]. In this study, they found that the mortality rates calculated with EuroSCORE were compatible with each other. In another study involving 668 patients, Tiras et al. reported that the logistic mortality rates predicted by the EuroSCORE (mortality sensitivity 80.95%) were close to the mortality and can be used safely [11]. In our study, in the preoperative evaluation, it was seen that the patients in Group 1 were mostly in the middle-risk group and the patients in Group 2 were in the low-risk group according to this scoring system, which predicted the mortality. These findings show that the EuroSCORE score is a preoperative parameter that supports our study by the fact that the expected mortality of Group 1, which included patients with low hematocrit levels, is higher than in Group 2, which included patients with normal hematocrit levels.

The most important perioperative factors affecting mortality and morbidity in CABG surgery are the number of grafts used, if greater than 4, cross-clamp time over 90 minutes, and CPB time over 120 minutes. Madhavan et al. reported that prolonged CPB time can cause postoperative complications, especially mortality [12]. And also in another study involving 3,799 patients who had cardiac surgery, prolonged cross-clamp time was reported to be significantly associated with postoperative morbidity and mortality [13]. In our study, there was no statistically significant difference between the two groups in terms of number of grafts used, cross-clamp time, CPB time, need for IABP support and AF development. Patients with hemodynamic instability were not included in the groups because of the difficulty in achieving a balanced distribution between preoperative variables and demographic data among the patient groups. This may be interpreted as the reason why in our study the perioperative process variables did not have statistical significance among the groups. The fact that there was no statistically significant difference in the number of grafts used, CPB time, and cross-clamp time revealed that there is no difference between the two groups in terms of CABG surgery.

There are many studies investigating the relationship between blood and blood product transfusions in patients undergoing cardiac surgery. Surgenor et al. reported that 36% (3254 patients) of 9,079 patients who underwent cardiac surgery received 1 to 2 units of erythrocyte transfusion, 43% of them were administered intraoperatively, the rest were administered postoperatively, and the mortality rate in patients receiving a transfusion was 16% higher than those who did not [14]. When 15,000 patients who underwent CPB at the Cleveland Clinic were examined, the relationship between transfusion and postoperative infections was assessed. It was reported that the frequency of septicemia, bacteremia, superficial, and deep wound infections had increased with erythrocyte use [15]. In a study conducted in England, 4,909 cardiac surgical patients who received transfusions were found to have longer hospitalization times and longer intensive care unit stays than 3,689 cardiac surgical patients who had not received a transfusion [16].

In our study, no significant differences were observed between the groups in terms of duration of postoperative mechanical ventilation, IABP support need, infection development, hospitalization time, and intensive care unit stay. However, there were significant differences between the two groups in terms of the amount of bleeding seen per the drainage systems and the number of blood and blood products needed. Although there is no significant difference in the number of reoperations between the groups in order to revise surgical bleeding, the amount of increased drainage in Group 1 became meaningful when there was an increase in the need for blood and blood products. Similar to the literature, although our study results were not statistically significant in Group 1, which included patients with low hematocrit values, the amount of drainage, use of blood products, infection development, length of intensive care follow-up, and length of hospital stay were higher than those in Group 2.

The preoperative low hematocrit level is independent of the need for a transfusion. The mortality rate increases when the need for a transfusion increases [17]. In a number of similar studies in recent years, preoperative low hematocrit levels in cardiac surgery have been shown to trigger postoperative complications [18,19]. Ranucci et al. examined 3,003 patients who underwent isolated CABG surgery. They found that patients with a preoperative hematocrit level of 33% or less developed a five-fold greater incidence of major morbidity than patients with a preoperative hematocrit level of 42% or more [20]. When the patients who underwent surgery were examined, it can be said that the CABG patients whose coronary reserves are already limited constitute the most sensitive patient group in the individuals that demonstrate a preoperative anemia [21,22].

In our study, the mortality rate in the patient group with low hematocrit levels was higher than the control group, which had normal hematocrit levels. This difference was statistically significant. The fact that the preoperative low hematocrit level affects negatively the postoperative processes and the first 30-day mortality rates of the patients is an indication of the need to be more careful in planning treatments for these patients. Even if the current mortality risk scoring systems do not include the hematocrit parameter, attention to the simple and routine hemogram test will help both the surgeon and the patient in terms of decision-making and planning the operation.

Limitations

This was a retrospective study on homogeneous patient groups. The limited number of patients was one of the limiting factors of the study. The operations performed by different surgeon groups and their results were examined. This makes standardization difficult. Our patients were analyzed in terms of in-hospital mortality and, therefore, medium and long-term results could not be evaluated. In addition, patients undergoing emergency surgery with preoperative hemodynamic instability, patients with a history of CABG, patients requiring additional surgery other than CABG, and patients with chronic renal failure were excluded. Therefore, the data of these critical patient groups were not included in the comparison. On the other hand, this situation prevented the critical preoperative situation from affecting the results of the study.

## Conclusions

Mortality rates after CABG surgery are still above the desired level and studies are underway to understand this situation and to reduce these rates. In our study, low preoperative hematocrit levels were associated with an increased mortality rate after CABG surgery. It is also a determinant of potentially receiving a postoperative blood transfusion, which has many risks and side effects. Therefore, detecting and treating the cause of low preoperative hematocrit levels may eliminate the undesirable consequences that may occur postoperatively. We believe that preoperative hematocrit levels should be added to the risk scoring systems, which could be used to evaluate the patients' postoperative mortality risk and to predict the length of hospital stay and cost-efficacy.
